# A Metataxonomic Tool to Investigate the Diversity of *Treponema*

**DOI:** 10.3389/fmicb.2019.02094

**Published:** 2019-09-10

**Authors:** Luisa K. Hallmaier-Wacker, Simone Lüert, Sabine Gronow, Cathrin Spröer, Jörg Overmann, Nicky Buller, Rebecca J. Vaughan-Higgins, Sascha Knauf

**Affiliations:** ^1^Neglected Tropical Diseases Work Group, Infection Biology Unit, German Primate Center, Leibniz Institute for Primate Research, Göttingen, Germany; ^2^Primate Genetics Laboratory, German Primate Center, Leibniz Institute for Primate Research, Göttingen, Germany; ^3^Leibniz Institute DSMZ – German Collection of Microorganisms and Cell Cultures, Braunschweig, Germany; ^4^Department of Microbiology, Braunschweig University of Technology, Braunschweig, Germany; ^5^Animal Pathology – Bacteriology Laboratory, Department of Primary Industries and Regional Development, South Perth, WA, Australia; ^6^Department of Conservation Medicine, College of Veterinary Medicine, School of Veterinary and Life Sciences, Murdoch University, Murdoch, WA, Australia

**Keywords:** metagenomics, metataxonomics, one health, spirochete, 16S rRNA, *Treponema*, marsupial, *Potorous*

## Abstract

The genus *Treponema* contains a number of human and animal pathogenic as well as symbiotic bacteria that are found in vastly different anatomical and environmental habitats. Our understanding of the species range, evolution, and biology of these important bacteria is still limited. To explore the diversity of treponemes, we established, validated, and tested a novel metataxonomic approach. As the informative nature of the hypervariable regions of the 16S rRNA gene differ, we first analyzed each variable region independently. Considering the *in silico* results obtained, we established and validated the sequencing of the V4-region of the 16S rRNA gene using known mixtures of *Treponema* species as well as a selected number of clinical samples. The metataxonomic approach was able to identify *Treponema* to a near-species level. We demonstrate that using a spirochete-specific enrichment, our method is applicable to complex microbial communities and large variety of biological samples. The metataxonomic approach described provides a useful method to unravel the full diversity and range of *Treponema* in various ecosystems.

## Introduction

*Spirochaetes*, a phylum of spiral-shaped bacteria, range from pathogenic (e.g., *Treponema pallidum*) to symbiotic (e.g., *Sphaerochaeta coccoides*) to free-living (e.g., *Exilispira thermophile*) species (Paster, 2001). The ability of spirochetes to inhabit vastly anatomical and ecological habitats is remarkable and indicates a high diversity of the bacterial members of this phylum (Paster, 2001). Until recently, spirochetes were predominantly discovered and subsequently characterized using cultivation, microscopical, or serological approaches. These techniques make it difficult and sometimes impossible to characterize not-yet-cultivated species, to identify species in multiple-spirochete infections, or to discover commensal microbes. The advent of cultivation-independent molecular techniques (e.g., nucleic acid amplification technology) has allowed for a broader detection of *Treponema* in different biological niches. To date, the 16S rRNA phylogenetic marker gene has been particularly instrumental in the detection of *Treponema* diversity (Pace, 1997). Based on defined similarity thresholds the 16S rRNA sequences can be grouped into phylotypes. For example, using a clonal 16S rRNA gene library and subsequent Sanger sequencing, the termite (*Reticulitermes flavipes*) gut was found to harbor more than 67 different treponemal phylotypes (Lilburn et al., 1999) and the human oral cavity up to 23 different treponemal clusters (Choi et al., 1994).

More recently, 16S rRNA gene-based metataxonomic studies have moved from clonal libraries to high-throughput sequencing approaches. Single hypervariable regions of the 16S rDNA have been used to examine different microbiomes (Kozich et al., 2013) and have identified treponemes in many ecological niches (Hong et al., 2012; Klitgaard et al., 2014; Rodriguez-R et al., 2015; Clayton et al., 2018; Hicks et al., 2018). For example, the gut microbiome of wild western lowland gorillas (*Gorilla gorilla gorilla*) (Hicks et al., 2018) and other nonhuman primates (Clayton et al., 2018) harbors multiple operational taxonomic units (OTUs) corresponding to the genus *Treponema*. Yet, conventional data analysis pipelines used in microbiome studies still do not allow for species-level characterization (Schloss et al., 2009; Caporaso et al., 2010). Taxonomic classification for many bacterial genera is restricted by the limited sequence differences in the 16S rRNA gene (Wang et al., 2007). Rossi-Tamisier et al. (2015) showed that spirochetes, in particular *Treponema* and *Spirochaeta*, have an exceptionally large variability in the 16S rRNA gene. For *Treponema*, only 2.1% of the analyzed 16S rRNA sequences fell within the recommended similarity threshold (95–98.7%) (Rossi-Tamisier et al., 2015).

To explore the range and diversity of *Treponema*, we established, validated, and tested a newly designed spirochete-specific metataxonomic approach that utilizes the 16S rRNA gene. Based on the known variability of the 16S rRNA gene, we hypothesized that a single hypervariable region of this gene provides a good target for a metagenomics-based assay to examine the diversity of *Treponema* in various biological sample types.

## Materials and Methods

### *In silico* Analysis

Based on the nomenclature of Bergey’s Manual of Systematic Bacteriology, we selected all bacterial species that are classified within the phylum *Spirochaetes* (Paster, 2001). Subsequently, a representative 16S rRNA gene sequence corresponding to each *Spirochaetes* bacterial species was retrieved from the GenBank database^1^. Where possible, sequences were chosen with maximal length and no ambiguous bases. Sequences shorter than 1,250 base pairs and/or containing more than two ambiguous bases were not included in the dataset even if no other sequence of the bacterial species was available (Supplementary Table S1).

The Perl-based high-throughput software tool V-Xtractor was used to locate the hypervariable regions (V2–V8) of the 16S rRNA sequences using Hidden Markov Models (Hartmann et al., 2010). Subsequently, the sequences of each variable region were analyzed using the mothur software package (v.1.41.1) (Schloss et al., 2009). In an initial step, identical sequences were removed using the unique.seq command. Then, the SILVA bacterial reference database (Quast et al., 2012) was utilized to align the sequences [align.seqs command using kmer searching (8mers) and Needleman–Wunsch pairwise alignment method]. OTU clustering was performed for distance threshold ranging from 0.01 to 0.10 at increments of 0.01 (cluster.split command with the OptiClust algorithm) (Westcott and Schloss, 2017).

### Spirochete Mock Community

The spirochete mock community comprised an equal mixture of 19 strains of the phylum *Spirochaetes*. Single bacterial DNA isolates were obtained from the German Collection of Microorganisms and Cell Cultures (DSMZ). DNA from rabbit inoculated *T. pallidum* subsp. *pertenue* strain Gauthier (referred to as *T. pallidum* throughout the manuscript) was obtained from David Šmajs, Department of Biology, Faculty of Medicine, Masaryk University, Brno, Czech Republic. The 19 *Spirochaetes* species which were used in this study, including the cultivation method, DSMZ reference number, 16S rRNA gene copy number, genome size, and NCBI reference, are shown in Supplementary Table S2.

The DNA of the cultured spirochetes obtained from DSMZ was quantified using the Qubit 2.0 Fluorometer (Thermo Fisher Scientific). *T. pallidum* DNA, due to the rabbit background DNA from *in vivo* inoculation experiments, was quantified using an established TaqMan PCR (qPCR) targeting the *polA* gene with slight modifications as described previously (Knauf et al., 2018). Based on the DNA content, genome size and 16S rRNA gene copy number, the 19 spirochetes were mixed together at equimolar (even) ribosomal RNA operon counts per organism. The final spirochete mock community contained 100,000 16S rDNA copies/μl of each species. All dilutions were made using Microbial DNA-Free water (Qiagen GmbH). Suitable precautions were taken during all sample handling and processing to avoid microbial contamination.

### *Treponema* Mock Communities

In addition to the spirochete mock community, we created three bacterial DNA validation sets to evaluate the intra-metagenomic assays performance. *T. pallidum* DNA was quantified using TaqMan PCR as described above. For the first validation set, the stock of *T. pallidum* (50,000 16S rRNA copies) was used to make a 10-fold dilution series. The dilutions of *T. pallidum* DNA were subsequently mixed with bacterial DNA contained no *Spirochaetes* [Microbial Mock Community, HM-280, Biodefense and Emerging Infectious Research (BEI) Resources, Manassas, VA, United States] (Supplementary Table S3). The second validation set was a mixture of *T. pallidum* and *T. denticola* in different ratios (Supplementary Table S4). The final ratios of the *T. pallidum* to *T. denticola* were 1:100, 1:10, 1:1, 10:1, and 100:1. The third validation set was a 10-fold serial dilution series of *T. pallidum* starting at 50,000 copies of 16S rRNA gene. Dilutions for all validation sets were made using Microbial DNA-Free water (Qiagen GmbH).

### Spirochete 16S Ribosomal RNA Gene Enrichment

Spirochete-selective primers were used to enrich spirochetal DNA (Dewhirst et al., 2010). The primers F24 (5′-GAGTTTGATYMTGGCTCAG-3′) and M98 (5′-GTTACGACTTCACCCYCCT-3′) were used to amplify a ∼1,450 bp fragment of the 16S rRNA gene covering the V1–V9 region. This first PCR step was performed in triplicates using the Phusion Hot Start II High-Fidelity DNA Polymerase (Thermo Fisher Scientific), which has been validated for the use in microbiome studies (Hallmaier-Wacker et al., 2018). PCR reactions consisted of 12.5 μl of 2× PCR master mix, 9.5 μl of Microbial DNA-Free water (Qiagen GmbH), 1.0 μl of each primer (0.5 mM each, Metabion), and 1 μl of template in a total reaction volume of 25 μl. PCR cycling conditions comprised of a pre-denaturation step of 30 s at 98°C, followed by either 20 or 35 cycles of 98°C for 10 s, 57°C for 15 s and 72°C for 120 s, and a final 10 min extension step at 72°C. A 16S rRNA amplification control sample (blank controls; Microbial DNA-Free water) was included. Subsequently, PCR triplicates were pooled before library preparation.

### Analysis of the V4 Region of the 16S rRNA Gene After an Initial Spirochete-Specific Amplification Step

A pre-test to re-amplify the V3, V4, and V6 regions was performed to identify the most suitable variable regions. The V4 region was selected, as the V3 and V6 region primers demonstrated technical issues to evenly amplify the variable regions. A modular, two-step PCR process was used to specifically re-amplify the V4-region of the 16S rRNA gene and prepare the samples for sequencing on the MiSeq platform. In the first step, the V4 region of the 16S rRNA gene was amplified using TruSeq adaptor-tailed universal primers 515F and 806R. The primers 515F-TruSeq (5′-ACACTCT TTCCCTCCACGACGCTCTTCCGCTCTGTGTGCCAGCMGC CGCGGTAA-3′) and 806R-TruSeq (5′-GTGACTGGAGTTCA GACGTGTGCTCTTCCGATCCCGGACTACHVGGGTWTCT AAT-3′) were composed of the universal primer targeting the V4 region (Caporaso et al., 2011) followed by a linker and the TruSeq adaptor (Illumina, Inc.). Amplification was performed in triplicates and each 25.0 μl reaction contained 1.0 μl of PCR product of the enrichment step, 12.5 μl of 2× Phusion Hot Start II High-Fidelity PCR Master Mix (Thermo Fisher Scientific), 9.5 μl of Microbial DNA-Free water (Qiagen GmbH), and 1.0 μl of each V4-targeting 16S primer (0.5 mM each, Metabion). The cycling conditions were as follows: a pre-denaturation step of 30 s at 98°C, followed by 20 cycles of 98°C for 10 s, 55°C for 15 s and 72°C for 60 s, and a final 10 min extension step at 72°C. To monitor contamination, the blank control of the enrichment step was included as a 16S rDNA amplification control.

In the second-step PCR reaction, sample-specific Illumina indices and flow cell adapters were added in an indexing reaction. Illumina i7 and i5 indices were added to each amplicon using the indexing primer P5 (5′-AATGATACGGCGACCACCGAG ATCTACAC-[i5-INDEX] -ACACTCTTTCCCTACACGACGCTC-3′) and indexing primer P7 (5′-CAAGCAGAAGACGGCATACGAGAT-[i7-INDEX]-GTGACTGGAGTTCAGAC GTGT-3′). Amplification was performed in a 50.0 μl reaction containing 2.0 μl of PCR product from the first-step, 25.0 μl of 2× KAPA HiFi HotStart ReadyMix (KAPA Biosystems), 21.0 μl of Microbial DNA-Free water (Qiagen GmbH), and 1.0 μl of each Truseq index primer (0.5 mM each, Metabion). The cycling conditions were as follows: a pre-denaturation step of 3 min at 98°C, followed by eight cycles of 98°C for 20 s, 62°C for 30 s and 72°C for 30 s, and a final 5 min extension step at 72°C. To monitor overall contamination, the blank control of the first-step PCR reaction was included as a 16S rRNA gene amplification control.

### V4-Region 16S rDNA Amplification Without an Initial Spirochete-Specific Amplification Step

For comparison the initial enrichment PCR was not performed on a sample of the spirochete mock community and a sample of validation set 1 (5,000 16S rDNA copies of *T. pallidum*). For these two samples the first-step V4-targeting PCR reaction was performed for additional 15 cycles (total of 35 cycles). A 16S rRNA gene amplification control was included for this altered procedure. All other conditions were kept identical.

### Applications to Clinical Samples

The applicability of the metataxonomic approach was tested on extracted DNA from genital swabs of Gilbert’s potoroo (*Potorous gilbertii*), a small marsupial found in Western Australia (Vaughan et al., 2009). For more information on sample processing see the Supplementary Materials.

### MiSeq Library Preparation and Pooling

After the indexing reaction, all amplicons were purified using 0.7× AMPure XP beads (Beckman Coulter), and quantified using the Qubit 2.0 Fluorometer (Thermo Fisher Scientific) (Supplementary Table S5). The amplicon integrity was verified for a representative number of four samples using the BioAnalyzer 2000 (Agilent). Equimolar amounts (2 nM) of sample amplicons were pooled. For samples with <2 nM concentration, the maximum volume (5 μl) was pooled prior to sequencing. The Transcriptome and Genome Analysis Laboratory at the University of Goettingen performed the Illumina MiSeq 2 × 250 bp paired-end sequencing (Illumina V2 chemistry) run.

### Data Processing and Analysis

Raw reads were processed using the mothur software package (version 1.41.1) (Schloss et al., 2009). Initial pre-processing and quality control were performed in accordance with the MiSeq SOP (Schloss et al., 2009). Briefly, paired-end reads were assembled using the make.contigs command. Subsequently, the screen.seqs command was used to trim sequences and filter out any sequences with ambiguous base calls. Identical trimmed sequences (unique.seq command) were aligned (align.seqs command) to the SILVA bacterial reference database (Quast et al., 2012). Poorly aligned sequences, chimeras [chimera.uchime command; UCHIME algorithm (Edgar et al., 2011)], and other erroneous non-bacterial sequences (remove.lineage command) were removed. The remaining sequences were classified using a Bayesian classifier implemented in mothur and OTUs were assigned based on a distance threshold of 0.03.

For the species-level classification, *Treponema*-classified sequences were extracted from the dataset using the get.lineage command. Using the *Treponema* sequence data in the *in silico* fasta file (Supplementary Table S1), a database was created using the create.database command. Using this database, the taxonomy of the filtered sequences was assigned using the classify.otu command.

### Data Availability

All generated read files have been deposited in the NCBI Sequence Read Archive under the accession number PRJNA541286.

## Results

### *In silico* Analysis of the Information Content of the V2–V8 Regions of the 16S rRNA Gene

We analyzed each hypervariable region (V2–V8) of the 16S rRNA gene for its potential to distinguish nine bacterial genera that make up the phylum *Spirochaetes*. In total, we analyzed the information content of the variable regions of 114 representative sequences *in silico* (Supplementary Table S1). Hypervariable regions V2–V8 were able to distinguish the nine bacterial genera at a similarity threshold of 97% (Table 1). The V2 region identified the largest total number of OTUs (*n* = 69) compared to all other tested regions (Table 1). Overall, the least number of OTUs were identified in the genera *Leptospira*, *Borrelia*, and *Brachyspira*. For the genus *Treponema* on the other hand, all variable regions with the exception of V7 were able to detect a high number of distinct OTUs (Table 1). Regions V2 and V3 were both able to detect 25 OTUs at a threshold of 97% in the *in silico* dataset containing 28 unique representative sequences. To examine the robustness of the *in silico* results for *Treponema*, we examined the identifiable OTUs at threshold cutoffs ranging from 90 to 99% (Supplementary Figure S1). For V2, V3, and V4 regions at 90% similarity threshold, >15 OTUs are distinguishable in the *Treponema* genus (Supplementary Figure S1).

**TABLE 1:** Identifiable *in silico* OTUs for the different genus within the phylum of *Spirochaetes* at a 97% threshold.

**Variable region**	**Total OTU**	***Treponema* (*n* = 28)^#^**	***Sphaerochaeta* (*n* = 4)**	***Spirochaeta* (*n* = 16)**	***Leptospira* (*n* = 21)**	***Exilispira* (*n* = 1)**	***Leptonema* (*n* = 1)**	***Borrelia* (*n* = 30)**	***Brachyspira* (*n* = 14)**	***Spironema* (*n* = 1)**
V2	69	25	3	14	11	1	1	5	8	1
V3	50	25	2	12	4	1	1	3	1	1
V4	53	24	3	13	3	1	1	4	3	1
V5	47	22	3	12	2	1	1	2	3	1
V6	53	24	4	12	6	1	1	2	3	1
V7	34	12	2	11	3	1	1	2	1	1
V8	47	23	2	11	5	1	1	1	2	1

*^#^(*n*) indicates the number of unique representative sequences in the *in silico*.fasta file for each genus.*

### V4-Region 16S rDNA Amplification of the Spirochete Mock Community

**FIGURE 1:**
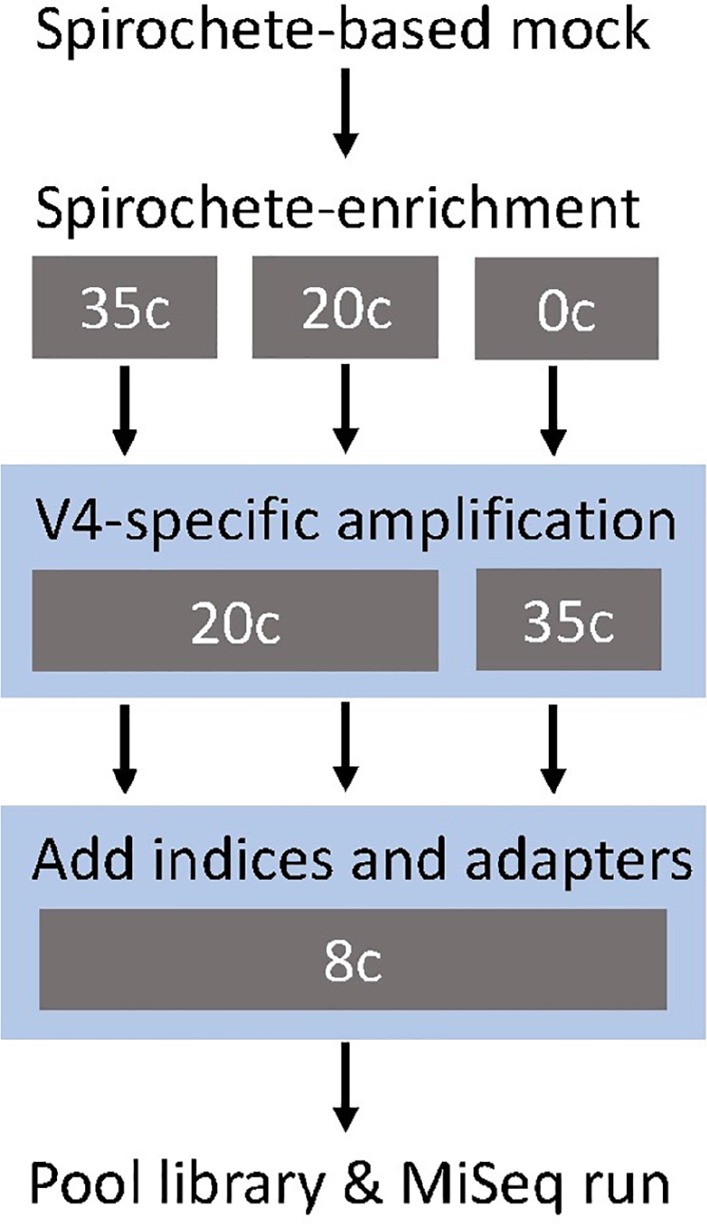
Study design of the metataxonomic assay targeting the V4-region of the 16S rRNA gene. The gray boxes show the tested cycle (c) conditions for each step. The blue shading indicates the modular two-step library preparation.

**FIGURE 2:**
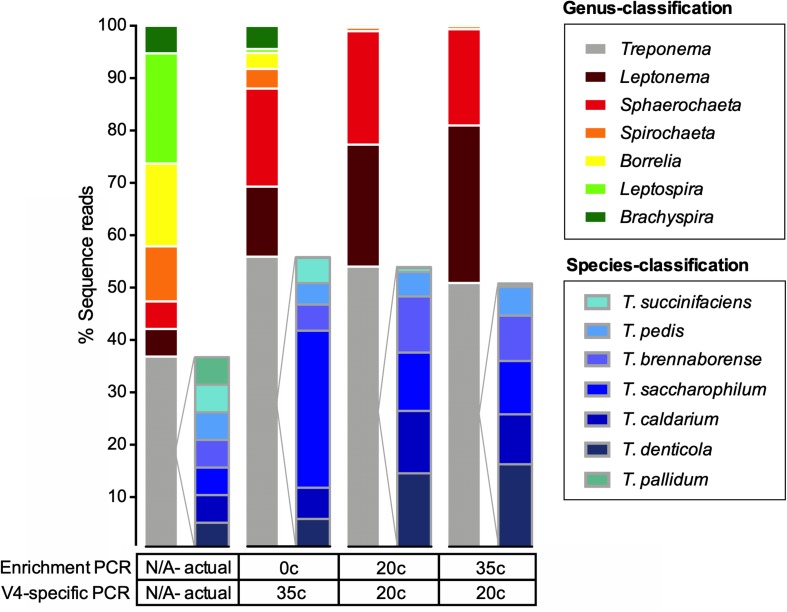
Actual and observed proportions of a spirochete mock community to a genus and species-level. Relative abundance of OTUs in percentage of reads for a spirochete microbial mock community amplified using multiple methods. The spirochete enrichment and V4-region-specific PCR cycle numbers are shown below the taxa plots. The expected strain proportion (actual) of the spirochete mock community represents the theoretical composition and was corrected for differential copy number of the 16S rRNA gene. Species-level classification only shown for the genus *Treponema*.

We tested three different amplification conditions targeting the V4-region of the 16S rRNA gene (Figures 1, 2). All 16S rRNA gene amplification conditions were able to identify all seven genera that were included in the mock community samples (Figure 2). The amplification condition without spirochete-specific 16S rRNA gene enrichment differed less from the actual mixing proportion than the samples which were enriched (Figure 2). In all conditions, *Treponema*, *Leptonema*, and *Sphaerochaeta* were preferentially detected. For the spirochete enriched samples (20 cycles and 35 cycles), *Borrelia*, *Leptospira*, and *Brachyspira* made up <1% of the detected sequence reads (Figure 2). In addition to the genus-level identification, we classified the *Treponema* sequences on a species-level using a *Treponema*-specific database of the V4-region. At 97% similarity threshold, the database contains 24 OTUs of which 21 OTUs correspond to single species and 3 OTUs correspond to species-clusters (*denticola*-, *medium*-, and the *pallidum*-cluster) (Supplementary Table S6). All 16S rDNA amplification conditions were able to identify all seven species of *Treponema* in the mock community (Figure 2). Independent of amplification conditions, sequences corresponding to *T. pallidum*-cluster were amplified less efficiently.

Amplification control samples (blank samples) were included for each amplification method to test for contamination during the amplification process. The blank sample from the 20-cycle enrichment had the lowest amplicon quantity before sequencing as well as the lowest overall corresponding number of sequences reads (Supplementary Figure S2 and Supplementary Table S5). Compared to the blank control enriched for 20 cycles, the control of the enrichment for 35 cycles had a 100× fold increase in sequence reads (Supplementary Figure S2). Despite an overall lower cycle count, the sequence reads corresponding to the nonenriched sample were as high as for the control enriched for 35 cycles. Unlike the enriched samples (20 cycle and 35 cycle), which detected minimal *Treponema* in the blank sample (<10 sequence reads), the non-enriched control sample detected 6,364 reads of *Treponema* (Supplementary Figure S2).

### Intra-Metagenomic Assays Performance

**FIGURE 3:**
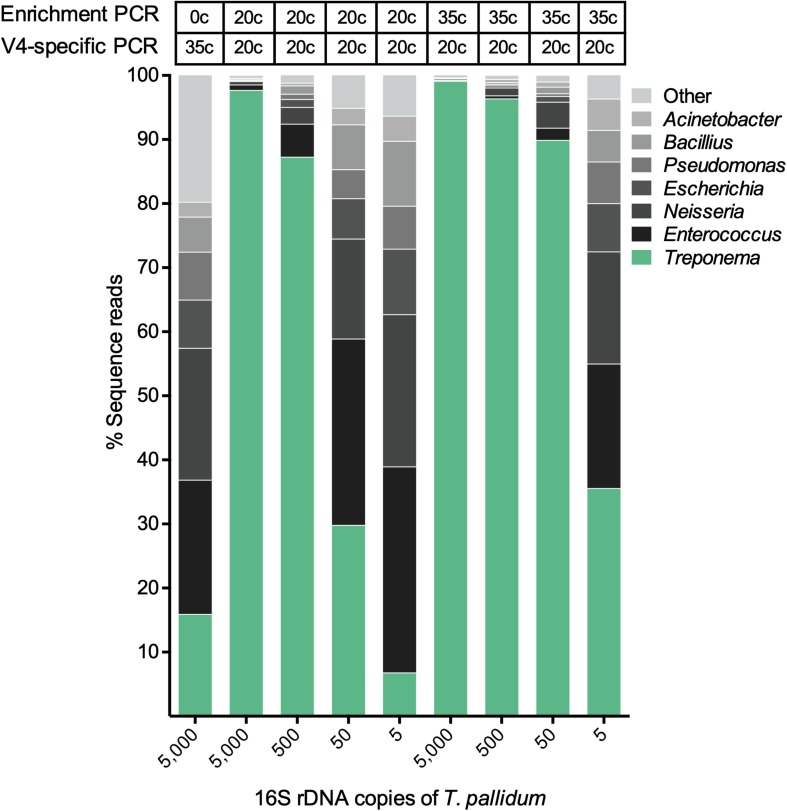
The effect of spirochete enrichment on the detection limit of *T. pallidum*. Relative abundance of OTUs in percentage of reads for a *T. pallidum*-spiked microbial mock community (HM-280). The theoretically calculated amount of spike in *T. pallidum* ranging from 5 to 5,000 16S rDNA copies is shown along the *x*-axis. The biologically effective range of *T. pallidum* may differ from theoretically calculated amount (e.g., 5 copies = 4–6 copies). Cycle number for the spirochete enrichment and V4-region-specific PCR is shown above the taxa plots.

The validation sample sets were used to assess the efficiency of the spirochete enrichment amplification, the effect of competition between two species, and the detection limit of the amplicon sequencing approach. The first validation set was a mixture of different concentrations of *T. pallidum* with a microbial mock community (HM-280). Figure 3 shows that both 20 cycle- and 35 cycle-enrichment steps significantly improve the detection of *T. pallidum* compared to the unenriched sample at 5,000 16S rRNA gene copies of *T. pallidum*. As the input DNA of *T. pallidum* decreases, the dilution effect between *T. pallidum* to microbial mock community HM-280 can be visualized clearly (Figure 3). Four to six 16S rRNA gene copies of *T. pallidum* were detected for 35 cycle- (44,141 sequence reads) and 20 cycle-enrichment (2,224 sequence reads).

**FIGURE 4:**
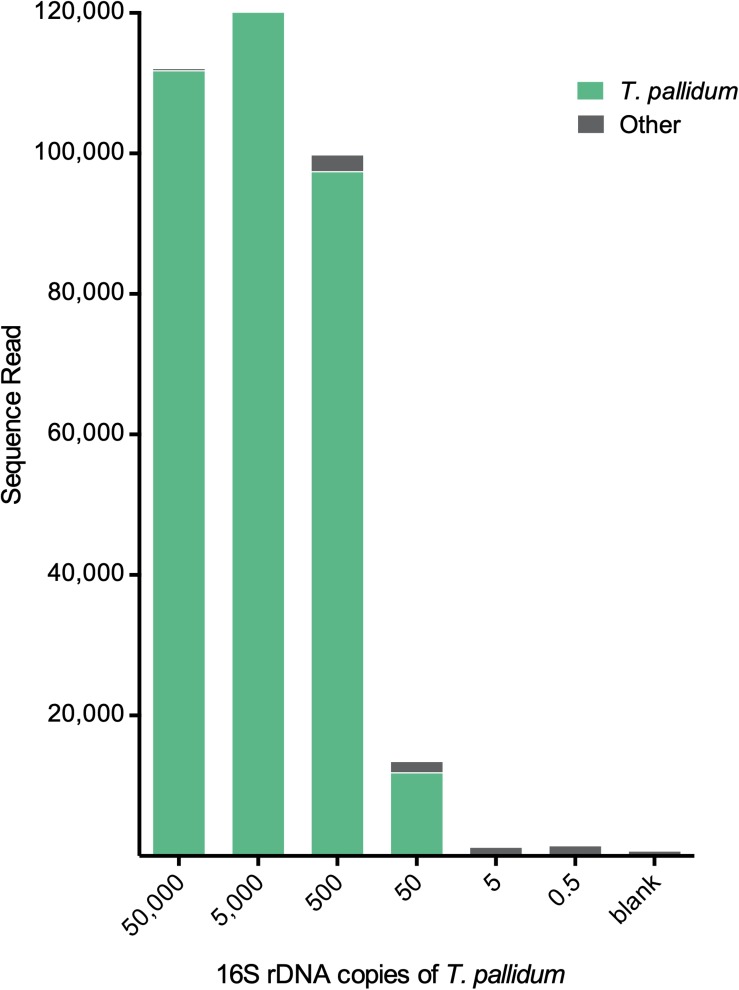
Detection limit of the metagenomic approach for *Treponema*. Total sequence reads resulting from different input amounts of *T. pallidum.* Displayed amounts of *T. pallidum* (50,000–0.5 16S rDNA copies) represent theoretically calculated amounts. Biologically effective range may differ from theoretically calculated amount (e.g., 0.5 copies = 0–2 copies). Blank control represents the 16S rDNA amplification control using microbial DNA-free water as input. For these samples 20 cycles of enrichment PCR was followed by 20 cycles of V4-region-specific PCR (for more detail see the section “Materials and Methods”).

The second validation set was a mixture of *T. pallidum* and *T. denticola* in different ratios (see the section “Materials and Methods” for details). For all ratios, *T. denticola* outcompeted *T. pallidum* in detected sequence reads (Table 2). However, both species of *Treponema* were detected at all tested ratios (Table 2). The third validation set was a 10-fold serial dilution series of *T. pallidum.* Sequence reads were >9,000 reads down to 500 16S rRNA gene copies of *T. pallidum* (Figure 4). At 50 16S rRNA gene copies, *T. pallidum* was detectable but overall sequence reads were markedly decreased (13,453 sequence reads). For the final two dilutions, total read numbers were <1,500 and *T. pallidum* sequence detection was analogous to the blank control (<10 sequence reads) (Figure 4).

**TABLE 2:** Relative abundance of OTUs in percentage of reads for different proportions of *T. pallidum* and *T. denticola* 16S rRNA gene.

**Ratio of *T. pallidum* and *T. denticola* 16S rRNA gene**	**% sequence reads for *T. pallidum* (read count)**	**% sequence reads for *T. denticola* (read count)**
1:100	0.1 (37)	99.9 (81,032)
1:10	0.3 (350)	99.7 (112,402)
1:1	3.0 (3,108)	97.0 (100,507)
10:1	26.6 (31,077)	73.4 (85,750)
100:1	77.3 (74,179)	22.7 (21,809)

### Applications to Clinical Samples (Gilbert’s Potoroo)

We examined samples from four Gilbert’s potoroo which had been found to harbor a *Treponema* infection (Vaughan et al., 2009). Using the amplicon sequencing technique, we identified a *Treponema* species in all four clinical samples (Supplementary Figure S3). Sequence reads corresponding to the *Treponema* made up >75% of the total read count for Gilbert’s potoroo samples No. 2–4 (Supplementary Figure S3). The *Treponema* sequences clustered into a single OTU, which cannot be identified using the *Treponema*-specific V4-region database at a 97% threshold identity.

## Discussion

We used the *in silico* analysis to predict the informative nature of each 16S rDNA hypervariable region for different spirochetes. Our findings expand on the results of Rossi-Tamisier et al. (2015), indicating that three genera of spirochetes have low interspecies sequence similarity with the 16S rRNA gene, which makes it a suitable gene target for identification (Table 1). The V3 and V4 region have been previously described for their discriminatory power (Chakravorty et al., 2007; Yang et al., 2016; Graspeuntner et al., 2018). In a study of 110 bacterial species, V2 and V3 were the most suitable candidates (Chakravorty et al., 2007). Considering phylogenetic resolution, the variable regions 4, 5, and 6 have been previously identified as prime targets (Yang et al., 2016; Graspeuntner et al., 2018). Overall, the *in silico* analysis provided good initial data to efficiently design the *in vitro* experiments. It is, however, important to note that technical caveats of NGS sequencing must be considered prior to the selection of the most appropriate region (Kozich et al., 2013). For example, for the Illumina MiSeq Platform, paired-end sequencing can currently cover 300 base pairs. Considering the overall error rate of this platform [∼0.1–0.01% per base, depending on the data-filtering scheme (Meacham et al., 2011; Loman et al., 2012)], the ideal read length for a metataxonomic approach allows for full overlap of the two pair-end reads (Kozich et al., 2013). Based on our *in silico* results and pre-test using different primers, we selected the V4 region of the 16S rRNA gene for further *in vitro* testing.

A spirochete mock community of known species composition allowed for the systematic comparison between the different amplification methods (Figure 1; Brooks et al., 2015). Independent of the amplification method, our metagenomic approach was able to detect all seven genera of spirochetes in the mock community, as well as all species of *Treponema* (Figure 2). However, not all spirochetes were detected equally well with all amplification procedures (Figure 2). The spirochete-specific enrichment step, which was included for a better detection of spirochetes, led to the distortion of the actual proportions and favored *Treponema*, *Sphaerochaeta*, and *Leptonema* (Figure 2). The distortion of the bacterial profiles due to preferential amplification of multi-template PCR is a known phenomenon and a major limitation of 16S rRNA gene amplification that results from sub-optimal primer binding (Polz and Cavanaugh, 1998; Brooks et al., 2015; Hallmaier-Wacker et al., 2018). It has been previously shown that this distortion effect is not significantly influenced with decreasing the number of amplification cycles (Acinas et al., 2005; Sipos et al., 2007; Wu et al., 2010; Brooks et al., 2015). Similarly, our results did not remarkably change with an increased number of enrichment cycles (20 cycles vs. 35 cycles; Figure 2). Nevertheless, the use of unnecessary cycles should be avoided as it can lead to formation of unwanted side products such as chimeras (Ahn et al., 2012), as well as a higher risk of overamplifying reads that originate from contamination (blank controls; Supplementary Figure S2) (Salter et al., 2014).

To examine the benefits of the spirochete enrichment PCR (20 cycles and 35 cycles) on the detection limit of analysis, we tested the metataxonomic approach on mock communities that simulate bacterial proportions found in clinical samples (Supplementary Table S3). For these samples, the enrichment step critically improved the detection of *Treponema* at low copy numbers, thus indicating that enrichment is a useful tool for samples with low spirochete numbers (<5,000 16S rDNA copies) (Figure 3). We showed that five 16S rRNA gene copies of *T. pallidum* were detectable in a sample with 20 other bacterial species (even bacterial mock HM-280). Using serial dilutions, we were able to detect as little as 50 16S rRNA gene copies of *T. pallidum* using 20 cycle enrichment amplification (Figure 4). These data indicate a sensitivity of our assay that is comparable to standard TaqMan qPCR and which outcompetes the conventional 16S rRNA clonal approach (Leslie et al., 2007). Obtaining a high detection limit using a clonal approach is both time consuming and resource intensive (Leigh et al., 2010). On the other hand, Sanger sequence analysis of clone libraries provide greater phylogenetic resolution due to an increased read length, covering the full 16S rRNA gene (Leigh et al., 2010). The complex microbial communities present in many clinical samples is a frequent challenge in diagnostics and in these sample the occurrence of multi-*Treponema* species is not uncommon. For example, in oral syphilis patients, *T. pallidum* can be found in combination with *T. denticola* (Scott and Flint, 2005). We therefore tested the effect of competing species by simulating a co-infection of *T. pallidum* and *T. denticola* (Supplementary Table S4). Overall, the metataxonomic approach underestimated the ratio of *T. pallidum* in the samples (Table 2). Amplicon sequencing was, however, sufficient to identify both species of *Treponema* at all tested ratios (Table 2). We note here that the metataxonomic approach does not accurately represent absolute abundance of different species (Widder et al., 2016). The used primers may have a significant effect in distorting tested ratios and thus alternative primer should be designed and evaluated for specific research questions. Additionally, quantitative techniques such as qPCR, flow cytometry, or fluorescence *in situ* hybridization (FISH) may be superior for evaluating known competing species (e.g., *T. pallidum and T. denticola*) (Props et al., 2017). Moreover, the metataxonomic approach should not be used for defining novel bacterial species even if species-level clustering is possible using the 16S rRNA sequence information (e.g., *Treponema*) (Tindall et al., 2010). Instead, 16S rRNA gene amplicon sequencing provides a qualitative view on the diversity of treponemes within a given DNA sample. For example, we used the metataxonomic approach to examine clinical samples of the Gilbert’s potoroo that have been previously found to harbor a *Treponema* infection (Vaughan et al., 2009). As the potoroo clinical samples were associated with a polymicrobial environment and infection was believed to be chronic (Vaughan et al., 2009), we performed a 35 cycle-enrichment in order to detect low concentrations of spirochetes. We identified a single *Treponema* species in all tested samples of the four Gilbert’s potoroos, which currently remains unclassified at a species level. The high percentage of *Treponema* in the detected samples (>75%; Supplementary Figure S3) indicates that the amplicon method is applicable for clinical samples and guides subsequent approaches that aim to fully characterize the discovered *Treponema* species. The results from the metataxonomic approach can be used to select most promising samples for whole-genome analysis (WGS), as well as provide a preliminary understanding of the possible phylogeny, which may assist in reference-based assembly (Wyres et al., 2014). Importantly, further WGS sequences of known and unknown *Treponema* are crucial to study the evolution and epidemiology of this ancient group of bacteria and to enhance future shotgun metagenomic studies. Currently, there is only a limited number of whole-genome sequences of *Treponema*, in particular the non-pathogenic species, due to the difficulty to culture many of the species [e.g., from the termite gut (Paster et al., 1996)].

## Conclusion

We showed that the V4 region of the 16S rRNA gene is a valuable target to explore the diversity of *Treponema* in various biological sample types. To monitor the quality of each sequencing run, it is essential to including relevant controls with all clinical samples. When applied appropriately, the presented modular metataxonomic approach is broadly applicable as it requires only small amounts of bacterial DNA for the detection of a broad range of *Treponema* species.

## Data Availability

The datasets generated for this study can be found in the NCBI Sequence Read Archive accession number PRJNA541286.

## Author Contributions

LH-W and SK conceived and designed the study. LH-W, SL, SG, CS, and SK performed the experiments in the laboratory. LH-W, SL, and SK analyzed the data. NB and RV-H contributed DNA from the Gilbert’s potoroos. SG, CS, and JO contributed DNA samples of spirochetes for the mock sample. All authors contributed to the writing of the manuscript, read, reviewed, and approved the final manuscript.

## Conflict of Interest Statement

SG, CS, and JO were employed by the Leibniz Institute DSMZ – German Collection of Microorganisms and Cell Cultures. The remaining authors declare that the research was conducted in the absence of any commercial or financial relationships that could be construed as a potential conflict of interest.

## Footnotes

1 https://www.ncbi.nlm.nih.gov
