# Corrigendum: A Metataxonomic Tool to Investigate the Diversity of *Treponema*

**DOI:** 10.3389/fmicb.2019.02581

**Published:** 2019-11-08

**Authors:** Luisa K. Hallmaier-Wacker, Simone Lüert, Sabine Gronow, Cathrin Spröer, Jörg Overmann, Nicky Buller, Rebecca J. Vaughan-Higgins, Sascha Knauf

**Affiliations:** ^1^Neglected Tropical Diseases Work Group, Infection Biology Unit, German Primate Center, Leibniz Institute for Primate Research, Göttingen, Germany; ^2^Primate Genetics Laboratory, German Primate Center, Leibniz Institute for Primate Research, Göttingen, Germany; ^3^Leibniz Institute DSMZ – German Collection of Microorganisms and Cell Cultures, Braunschweig, Germany; ^4^Department of Microbiology, Braunschweig University of Technology, Braunschweig, Germany; ^5^Animal Pathology – Bacteriology Laboratory, Department of Primary Industries and Regional Development, South Perth, WA, Australia; ^6^Department of Conservation Medicine, College of Veterinary Medicine, School of Veterinary and Life Sciences, Murdoch University, Murdoch, WA, Australia

**Keywords:** metagenomics, metataxonomics, one health, spirochete, 16S rRNA, *Treponema*, marsupial, *Potorous*

In the original article, there was a mistake in Figure 1 as published. The figure contains an error in the number of cycles for the “add indices and adapters” step. The step should read 8 cycles instead of 20 cycles. The corrected Figure 1 appears below.

**Figure 1:**
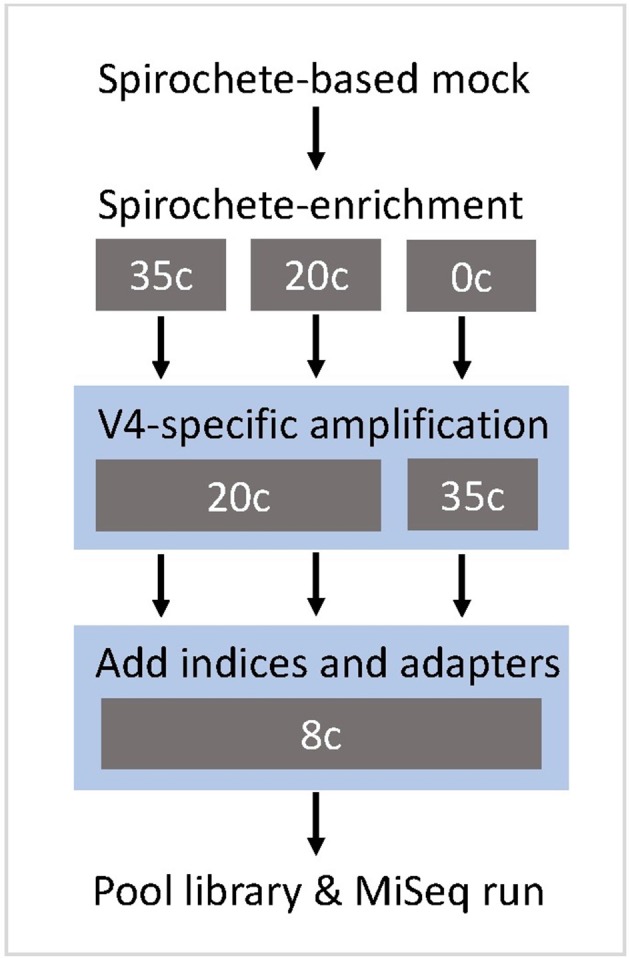
Study design of the metataxonomic assay targeting the V4-region of the 16S rRNA gene. The gray boxes show the tested cycle (c) conditions for each step. The blue shading indicates the modular two-step library preparation.

The authors apologize for this error and state that this does not change the scientific conclusions of the article in any way. The original article has been updated.

