# Supplementary material for: A Metataxonomic Tool to Investigate the Diversity of Treponema
Source: Front Microbiol. 2019 Sep 10;10:2094. doi: 10.3389/fmicb.2019.02094 (PMC6746968; doi:10.3389/fmicb.2019.02094)
Supplement: Supplementary file 1 [file Data_Sheet_1.docx]

**A metataxonomic tool to investigate the diversity of *Treponema***

Luisa K. Hallmaier-Wacker^1,2^, Simone Lüert^1,2^, Sabine Gronow^3^, Cathrin Spröer^3^, Jörg Overmann^3,4^, Nicky Buller^5^, Rebecca J. Vaughan-Higgins^6^, Sascha Knauf^1*^

***Supplementary Materials***

**1 Supplementary Material and Methods**

***Processing of Gilbert´s potoroo samples.*** We obtained previously extracted DNA from clinical samples that were taken for a different study (1). The collection of the samples was reviewed and approved by the Murdock University Animal Ethics Committee (W1082/04). No animals were handled for this study. Please refer to Vaughan-Higgins *et al*. for details on sampling procedures and DNA extraction. The metataxonomic assay was performed for the Gilbert´s potoroo samples using 35-cycles enrichment PCR followed by two-step Truseq library preparation. Data analysis including species-level classification for *Treponema* was performed as described for the mock communities.

**2 Supplementary Tables**

**Table S1.** Spirochetes (with and without taxonomic validation*) and corresponding NCBI Accession numbers used in *in silico* determination. *The taxonomic standing of each spirochete species can be cross-checked using the NCBI GenBank Accession Number and Integrated Taxonomix Information System.

| **Genus** | **Species** | **GenBank Number** |
| --- | --- | --- |
| *Borrelia* | *afzelii* | JX888452 |
| *Borrelia* | *americana* | NR_116166 |
| *Borrelia* | *anserina* | U42284.1 |
| *Borrelia* | *bavariensis* | NR_074854 |
| *Borrelia* | *burgdorferi* | AB091823 |
| *Borrelia* | *carolinensis* | EU085416 |
| *Borrelia* | *coriaceae* | NR_114544 |
| *Borrelia* | *crocidurae* | GQ358200 |
| *Borrelia* | *duttonii* | AF107366 |
| *Borrelia* | *garinii* | D67018 |
| *Borrelia* | *hermsii* | EU203150 |
| *Borrelia* | *hispanica* | GQ202264 |
| *Borrelia* | *japonica* | L46696 |
| *Borrelia* | *lonestari* | AY166715 |
| *Borrelia* | *mayonii* | KP972468 |
| *Borrelia* | *miyamotoi* | AB904793 |
| *Borrelia* | *parkeri* | AF307100 |
| *Borrelia* | *persica* | HQ610931 |
| *Borrelia* | *recurrentis* | AF107361 |
| *Borrelia* | *sinica* | NR_024713 |
| *Borrelia* | *spielmanii* | AM182231 |
| *Borrelia* | *tanukii* | D67023 |
| *Borrelia* | *theileri* | KF569941 |
| *Borrelia* | *turcica* | NR_024820 |
| *Borrelia* | *turdi* | D67024 |
| *Borrelia* | *turicatae* | AY934610 |
| *Borrelia* | *valaisiana* | EU135596 |
| *Borrelia* | *yangtzensis* | NR_145665 |
| *Brachyspira* | *aalborgi* | AF200693 |
| *Brachyspira* | *alvinipulli* | JF430707 |
| *Brachyspira* | *canis* | HM450994 |
| *Brachyspira* | *corvi* | EU819070 |
| *Brachyspira* | *hyodysenteriae* | NR_044764 |
| *Brachyspira* | *innocens* | NR_044763 |
| *Brachyspira* | *intermedia* | KR809388 |
| *Brachyspira* | *murdochii* | KR809386 |
| *Brachyspira* | *muridarum* | GU189376 |
| *Brachyspira* | *muris* | GU189383 |
| *Brachyspira* | *pilosicoli* | AB120008 |
| *Brachyspira* | *pulli* | KR809387 |
| *Brachyspira* | *rattus* | GU189374 |
| *Brachyspira* | *suanatina* | DQ473578 |
| *Exilispira* | *thermophila* | NR_041644 |
| *Leptonema* | *illini* | JQ988853 |
| *Leptospira* | *alexanderi* | JQ988836.1 |
| *Leptospira* | *biflexa* | JQ988840.1 |
| *Leptospira* | *borgpetersenii* | AY995716 |
| *Leptospira* | *broomii* | Y19243 |
| *Leptospira* | *fainei* | JQ988851.1 |
| *Leptospira* | *idonii* | AB721966.1 |
| *Leptospira* | *inadai* | AY631896.1 |
| *Leptospira* | *interrogans* | AY995726 |
| *Leptospira* | *kirschneri* | EF536998 |
| *Leptospira* | *kmetyi* | AB279549.1 |
| *Leptospira* | *licerasiae* | NR_044310 |
| *Leptospira* | *mayottensis* | NR_134067.1 |
| *Leptospira* | *meyeri* | HQ709385.1 |
| *Leptospira* | *noguchii* | EU349496 |
| *Leptospira* | *santarosai* | JQ988838.1 |
| *Leptospira* | *terpstrae* | NR_115294.1 |
| *Leptospira* | *vanthielii* | NR_115297.1 |
| *Leptospira* | *weilii* | JQ988839.1 |
| *Leptospira* | *wolbachii* | AY631879.1 |
| *Leptospira* | *wolffii* | KC662454.1 |
| *Leptospira* | *yanagawae* | MG979779.1 |
| *Sphaerochaeta* | *associata* | NR_145842.1 |
| *Sphaerochaeta* | *coccoides* | NR_042260 |
| *Sphaerochaeta* | *globosa* | NR_114608 |
| *Sphaerochaeta* | *pleomorpha* | NR_114609 |
| *Spirochaeta* | *africana* | NR_026302 |
| *Spirochaeta* | *alkalica* | NR_026301 |
| *Spirochaeta* | *americana* | NR_028820 |
| *Spirochaeta* | *asiatica* | NR_026300 |
| *Spirochaeta* | *aurantia* | FR749896.1 |
| *Spirochaeta* | *cellobiosiphila* | NR_044505 |
| *Spirochaeta* | *dissipatitropha* | AY995150.1 |
| *Spirochaeta* | *halophila* | NR_044756.2 |
| *Spirochaeta* | *isovalerica* | FR749931.1 |
| *Spirochaeta* | *lutea* | HG965770.2 |
| *Spirochaeta* | *perfilievii* | AY337318 |
| *Spirochaeta* | *psychrophila* | NR_134185.1 |
| *Spirochaeta* | *smaragdinae* | NR_027585 |
| *Spirochaeta* | *taiwanensis* | AY735103.1 |
| *Spirochaeta* | *thermophila* | CP002903.1 |
| *Spirochaeta* | *xylanolyticus* | AY735097 |
| *Spironema* | *culicis* | AF166259.1 |
| *Treponema* | *amylovorum* | JN713358 |
| *Treponema* | *azotonutricium* | NR_074168 |
| *Treponema* | *berlinense* | NR_042797.1 |
| *Treponema* | *brennaborense* | NR_029348 |
| *Treponema* | *bryantii* | AB849328 |
| *Treponema* | *caldarium* | NC_015732.1 |
| *Treponema* | *denticola* | KC415235 |
| *Treponema* | *isoptericolens* | NR_042486.1 |
| *Treponema* | *lecithinolyticum* | GU420631 |
| *Treponema* | *maltophilum* | X87140 |
| *Treponema* | *medium* | JN713397 |
| *Treponema* | *pallidum* | NC_021179 |
| *Treponema* | *paraluisleporidarum* ecovar Cuniculus | NC_015714 |
| *Treponema* | *paraluisleporidarum* ecovar Lepus | JX899416 |
| *Treponema* | *parvum* | AF302939.1 |
| *Treponema* | *pectinovorum* | GU562449.1 |
| *Treponema* | *pedis* | KP063170 |
| *Treponema* | *phagedenis* | FJ004921 |
| *Treponema* | *porcinum* | NR_042942 |
| *Treponema* | *primitia* | NC_015578 |
| *Treponema* | *putidum* | NR_027189 |
| *Treponema* | *saccharophilum* | M71238 |
| *Treponema* | *socranskii* | AB015892 |
| *Treponema* | *stenostreptum* | NR_113042 |
| *Treponema* | *succinifaciens* | NR_074755.1 |
| *Treponema* | *vincentii* | AY119690 |
| *Treponema* | *zioleckii* | DQ065758 |
| *Treponema* | *zuelzerae* | NR_104797 |

**Table S2.** Organisms included in the spirochete mock community. * Catalogue number of the German Collection of Microorganisms and Cell Cultures (DSMZ).

| DSM #* | Genus | Species | Cultivation | Genome size (Mbp) | 16S rDNA copies | NCBI Reference |
| --- | --- | --- | --- | --- | --- | --- |
| 10508 | *Borrelia* | *afzelii* | pure culture | 0.905 | 2 | NC_018887.1 |
| 5251 | *Borrelia* | *hermsii* | pure culture | 0.923 | 1 | NZ_CP014349.1 |
| 4680 | *Borrelia* | *burgdorferi* | pure culture | 0.911 | 1 | NC_001318.1 |
| 105803 | *Brachyspira* | *hyodysenteriae* | pure culture | 3.041 | 1 | NZ_CP015910.2 |
| 21528 | *Leptonema* | *illini* | pure culture | 4.521 | 1 | NZ_AHKT00000000.1 |
| 21526 | *Leptospira* | *kirschneri* | pure culture | 4.409 | 1 | NZ_AHMN00000000.2 |
| 21537 | *Leptospira* | *meyeri* | pure culture | 4.188 | 1 | NZ_AKXE00000000.1 |
| 21521 | *Leptospira* | *terpstrae* | pure culture | 4.092 | 2 | NZ_AOGW00000000.2 |
| 21534 | *Leptospira* | *broomii* | pure culture | 4.395 | 3 | NZ_AHMO00000000.2 |
| 22777 | *Sphaerochaeta* | *globosa* | pure culture | 3.316 | 4 | NC_015152.1 |
| 6578 | *Spirochaeta* | *thermophila* | pure culture | 2.560 | 2 | NC_017583.1 |
| 8902 | *Spirochaeta* | *africana* | pure culture | 3.286 | 3 | NC_017098.1 |
| 12168 | *Treponema* | *brennaborense* | pure culture | 3.056 | 4 | NC_015500.1 |
| 14222 | *Treponema* | *denticola* | pure culture | 2.843 | 2 | NC_002967.9 |
| 18691 | *Treponema* | *pedis* | pure culture | 2.889 | 2 | NC_022097.1 |
| 2985 | *Treponema* | *saccharophilum* | pure culture | 3.454 | 1 | NZ_AGRW00000000.1 |
| 2489 | *Treponema* | *succinifaciens* | pure culture | 2.732 | 4 | NC_015385.1 |
| 7334 | *Treponema* | *caldarium* | pure culture | 3.239 | 3 | NC_015732.1 |
| N/A | *Treponema* | *pallidum* | *in vivo* | 1.139 | 2 | NZ_CP003679.1 |

**Table S3.** Bacterial mixture of validation set 1. * Microbial mock community, HM-280 (Biodefense and Emerging Infectious Research (BEI) Resources, Manassas, USA) contains no *Spirochaetes*. ^#^ Effective biological range is 4-6 copies of 16S rDNA.

| Mock community HM-280 *  (ng/µl) | Concentration of *T. pallidum (*copies of 16S rDNA) | |
| --- | --- | --- |
| 0.50 | | 5,000 |
| 0.50 | | 500 |
| 0.50 | | 50 |
| 0.50 | | 5 ^#^ |

**Table S4.** Bacterial mixture of validation set 2.

| Concentration of *T. pallidum (*copies of 16S rDNA) | | Concentration of *T. denticola (*copies of 16S rDNA) | Final ratio of *T. pallidum* to *T. denticula* |
| --- | --- | --- | --- |
| 500 | 50,000 | | 1:100 |
| 5,000 | 50,000 | | 1:10 |
| 50,000 | 50,000 | | 1:1 |
| 50,000 | 5,000 | | 10:1 |
| 50,000 | 500 | | 100:1 |

**Table S5.** Qubit measurements of spirochete mock community, *Treponema* validation sets (Val1-3) and blank controls included in this study. * *TP*: theoretically computed *T. pallidum* 16Sr RNA gene copies in each sample; ^#^ OFR: out of range ≤ 0.2 ng/µl.

| Sample | Enrichment PCR (cycles) | V4-specific PCR (cycles) | Qubit Average (ng/µl) |
| --- | --- | --- | --- |
| Spirochete mock | 0 | 35 | 12.8 |
| Spirochete mock | 20 | 20 | 14.9 |
| Spirochete mock | 35 | 20 | 16.8 |
| Val1 (5,000 copies TP)* | 0 | 35 | 12.8 |
| Val1 (5,000 copies TP) | 20 | 20 | 2.5 |
| Val1 (500 copies TP) | 20 | 20 | 3.3 |
| Val1 (50 copies TP) | 20 | 20 | 0.43 |
| Val1 (5 copies TP) | 20 | 20 | 0.45 |
| Val1 (5,000 copies TP) | 35 | 20 | 9.4 |
| Val1 (500 copies TP) | 35 | 20 | 12.0 |
| Val1 (50 copies TP) | 35 | 20 | 10.6 |
| Val1 (5 copies TP) | 35 | 20 | 29.3 |
| Val2 (1:100) | 20 | 20 | 2.0 |
| Val2 (1:10) | 20 | 20 | 6.8 |
| Val2 (1:1) | 20 | 20 | 4.9 |
| Val2 (10:1) | 20 | 20 | 8.2 |
| Val2 (100:1) | 20 | 20 | 2.4 |
| Val3 (50,000 copies TP) | 20 | 20 | 3.4 |
| Val3 (5,000 copies TP) | 20 | 20 | 8.3 |
| Val3 (500 copies TP) | 20 | 20 | 1.7 |
| Val3 (50 copies TP) | 20 | 20 | 0.14 |
| Val3 (5 copies TP) | 20 | 20 | OFR^#^ |
| Val3 (0.5 copies TP) | 20 | 20 | OFR |
| Blank control | 0 | 35 | 1.1 |
| Blank control | 20 | 20 | OFR |
| Blank control | 35 | 20 | 0.3 |

**Table S6.** *In silico* results for identifiable OTUs at a 97% threshold for the V4-region of the 16S rRNA gene of the *Treponema* genus.

| OTU | Species association | OTU Representation |
| --- | --- | --- |
| 1 | *T. amylovorum* | Single species |
| 2 | *T. azotonutricium* | Single species |
| 3 | *T. berlinense* | Single species |
| 4 | *T. brennaborense* | Single species |
| 5 | *T. bryantii* | Single species |
| 6 | *T. caldarium* | Single species |
| 7 | *T. denticola, T. putidum* | *Denticola*-cluster |
| 8 | *T. isoptericolens* | Single species |
| 9 | *T. lecithinolyticum* | Single species |
| 10 | *T. maltophilum* | Single species |
| 11 | *T. medium, T. vincentii* | *Medium*-cluster |
| 12 | *T. pallidum, T. paraluisleporidarum* | *Pallidum*-cluster |
| 13 | *T. pectinovorum* | Single species |
| 14 | *T. pedis* | Single species |
| 15 | *T. parvum* | Single species |
| 16 | *T. phagedenis* | Single species |
| 17 | *T. porcinum* | Single species |
| 18 | *T. primitia* | Single species |
| 19 | *T. saccharophilum* | Single species |
| 20 | *T. socranskii* | Single species |
| 21 | *T. stenostreptum* | Single species |
| 22 | *T. succinifaciens* | Single species |
| 23 | *T. zioleckii* | Single species |
| 24 | *T. zuelzerae* | Single species |

**3 Supplementary Figures**

**Fig. S1*. In silico* results for identifiable OTUs at different threshold cut-offs for the *Treponema* genus.** Gray bar graphs show the number of OTUs detected at thresholds ranging from 99% to 90% similarity for 16S rDNA region (A) V2, (B) V3, (C) V4, (D) V5, (E) V6, (F) V7 and (G) V8. The dashed line on the plots represents the number of defined *Treponema* species in *in silico* dataset.

**Fig. S2. Bacterial profile of control samples included in this study.** Total sequence reads corresponding to 16S rRNA amplification control samples (blank controls) in this study. The spirochete enrichment and V4-region specific PCR cycle number are shown below the plot.

**Fig. S3. Sequence read counts of Gilbert´s potoroo samples.** Total sequence reads corresponding to four clinical samples. For the clinical samples, 35-cycles of spirochete enrichment PCR was followed by 20-cycles of V4-region specific PCR (for more detail see the methods).

**4 Supplementary References**

1. Vaughan RJ, Warren KS, Mills JS, Palmer C, Fenwick S, Monaghan CL, Friend AJ. 2009. Hematological and serum biochemical reference values and cohort analysis in the Gilbert's potoroo (*Potorous gilbertii*). J Zoo Wildl Med 40:276-288.
